# Inhibitory Effects of Macrotetrolides from *Streptomyces* spp. On Zoosporogenesis and Motility of Peronosporomycete Zoospores Are Likely Linked with Enhanced ATPase Activity in Mitochondria

**DOI:** 10.3389/fmicb.2016.01824

**Published:** 2016-11-18

**Authors:** Md. Tofazzal Islam, Hartmut Laatsch, Andreas von Tiedemann

**Affiliations:** ^1^Division of Plant Pathology and Plant Protection, Department of Crop Sciences, Georg-August-Universität GöttingenGöttingen, Germany; ^2^Department of Biotechnology, Bangabandhu Sheikh Mujibur Rahman Agricultural UniversityGazipur, Bangladesh; ^3^Institute of Organic and Biomolecular Chemistry, Georg-August-Universität GöttingenGöttingen, Germany

**Keywords:** ionophore, dinactin, zoosporocides, mitochondrial ATPase activity, biological control

## Abstract

The release of zoospores from sporangia and motility of the released zoospores are critical in the disease cycle of the Peronosporomycetes that cause devastating diseases in plants, fishes, animals and humans. Disruption of any of these asexual life stages eliminates the possibility of pathogenesis. In the course of screening novel bioactive secondary metabolites, we found that extracts of some strains of marine *Streptomyces* spp. rapidly impaired motility and caused subsequent lysis of zoospores of the grapevine downy mildew pathogen *Plasmopara viticola* at 10 μg/ml. We tested a number of secondary metabolites previously isolated from these strains and found that macrotetrolide antibiotics such as nonactin, monactin, dinactin and trinactin, and nactic acids such as (+)-nonactic acid, (+)-homonactic acid, nonactic acid methyl ester, homonactic acid methyl ester, bonactin and feigrisolide C impaired motility and caused subsequent lysis of *P. viticola* zoospores in a dose- and time-dependent manners with dinactin being the most active compound (MIC 0.3 μg/ml). A cation channel-forming compound, gramicidin, and a carrier of monovalent cations, nigericin also showed similar biological activities. Among all 12 compounds tested, gramicidin most potently arrested the motility of zoospores at concentrations starting from 0.1 μg/ml. All macrotetrolide antibiotics also displayed similar motility impairing activities against *P. viticola, Phytophthora capsici*, and *Aphanomyces cochlioides* zoospores indicating non-specific biological effects of these compounds toward peronosporomyctes. Furthermore, macrotetrolide antibiotics and gramicidin also markedly suppressed the release of zoospores from sporangia of *P. viticola* in a dose-dependent manner. As macrotetrolide antibiotics and gramicidin are known as enhancers of mitochondrial ATPase activity, inhibition of zoosporogenesis and motility of zoospores by these compounds are likely linked with hydrolysis of ATP through enhanced ATPase activity in mitochondria. This is the first report on motility inhibitory and lytic activities of macrotetrolide antibiotics and nactic acids against the zoospores of peronosporomycete phytopathogens.

## Introduction

The Peronosporomycete genera such as *Plasmopara, Phytophthora, Pythium*, and *Aphanomyces* are notorious pathogens of plants, fishes and vertebrates (Agrios, 1997). Although, morphologically and physiologically they have similarities to fungi, phylogenetically they are relatives of brown algae and diatoms and thus belong to the kingdom of Straminipila (Dick, 2001). One of the unique features of the peronosporomycete pathogens is that in favorable environment they asexually produce motile zoospores from sporangia that develop at the tip of branched sporangiophores (Judelson and Blanco, 2005). Sporangia are globose to lemon-shaped containers capable of converting their cytoplasm into multiple wall-less zoospores. The release of zoospores from sporangia (zoosporogenesis) involves cleavage of the sporangial cytoplasm by nucleus-enveloping membrane networks and an assembly of two flagellae per zoospore (Hardham and Hyde, 1997). The produced zoospores are expelled from the sporangium through sporangial papillae by turgor pressure, resulting in part from high concentration of proline that accumulates in the cytoplasm of cleaving sporangia (Ambikapathy et al., 2002). The zoospores swim after their release, using an anterior tinsel-type flagellum ornamented with tripartite tubular hairs to pull the cell and a posterior whiplash-type flagellum for steering that accomplish “thrust reversal” (Islam et al., 2002b; Judelson and Blanco, 2005). The primary goal of swimming zoospores is to find potential infection sites of the host guided by host-mediated signaling cues (Islam and Tahara, 2001), followed by morphological change into round cystospores by cellular encystment and shedding of flagellae (Islam et al., 2002b, 2003). The cystospores rapidly germinate to form hyphal germ tubes, which penetrate host tissues for infection. Although, little is known about the underlying molecular mechanisms of zoosporogenesis and motility pathways, it has been found that substantial energy from reserve β-1,3-glucan (mycolaminarin) is used during zoosporogenesis and for motility of zoospores (Bimpong, 1975; Judelson and Blanco, 2005). Zoospores are highly energy demanding life stages as ATPase activity in zoospores is similar to that in contracting skeletal muscles (Holker et al., 1993; Stienen et al., 1996). Inhibition of enzymes that maintain ATP concentration, or shuttling of ATP from mitochondria to sites of high utilization such as flagellar kinetosomes of zoospores or depletion of ATP by enhancement of ATPase activity would result in impairment of motility of zoospores and suppression of zoosporogenesis (Judelson and Blanco, 2005).

*Plasmopara viticola* is a devastating downy mildew pathogen of grapevine, which causes severe economic losses worldwide (Agrios, 1997). This pathogen spreads by an extremely efficient cycle of asexual propagation (Kiefer et al., 2002; Riemann et al., 2002). The success of this obligate biotrophic pathogen can be attributed in part to the speed of asexual differentiation to generate biflagellate motile zoospores from airborne sporangia (zoosporogenesis). It has been observed that zoospores locate the stomata being guided by biochemical host cues followed by encystment and germination to form germ tubes to initiate infection through stomata (Kiefer et al., 2002). Disruption of zoospore release from sporangia (zoosporogenesis) and/or motility of zoospores by any kind of inhibitor eliminates the potential for pathogenesis (Judelson and Blanco, 2005; Islam et al., 2011). Control of downy mildew in practice is difficult as host plant resistance is generally low and the polycyclic life style of the pathogen requires frequent treatments with fungicides. Novel biorational approaches may help to improve control of this notorious phytopathogen. Discovery of inhibitory chemical substances that can affect the pathways of zoosporogenesis and/or motility of zoospores might be useful for development of innovative and effective strategies for controlling the disease (Judelson and Blanco, 2005; Islam et al., 2011).

Due to the biotrophic nature, *P. viticola* is recalcitrant to cultivation on culture media and thus it is difficult to test the inhibitory potential of novel chemical compounds on zoospore release from sporangia or the motility of zoospores. We developed *in vitro* methods to produce high quantities of sporangia on excised grapevine leaves and get copious amounts of biflagellate motile zoospores in a host-free system (Islam and von Tiedemann, 2008). The released zoospores remain motile in sterilized water for 12–16 h. These *in vitro* methods allow screening secondary metabolites from antagonistic environmental microorganisms using convenient bioassay protocols (Islam et al., 2011). In this way, we recently isolated several new secondary metabolites from plants and microorganisms that suppress zoosporogenesis, inhibit motility and/or cause lysis of *P. viticola* zoospores (Abdalla et al., 2011; Islam et al., 2011; Zinad et al., 2011; Talontsi et al., 2012a,b; Dame et al., 2016). Furthermore, using a natural product, staurosporine and some further kinase inhibitors, we recently demonstrated that protein kinase C is involved in both flagellar motility and zoosporogenesis of the Peronosporomyces (Islam et al., 2011).

Secondary metabolites from marine microorganisms especially *Streptomyces* spp. are known to possess diverse biological activities through inhibiting specific enzyme or proteins in the signaling pathways (Islam et al., 2011). In the course of screening for novel secondary metabolites from marine *Streptomyces* spp., we found that extracts of some marine *Streptomyces* spp. (such as strains Act 8970, ACT 7619) rapidly impaired motility and caused subsequent lysis of zoospores at 10 μg/ml. We then tested all previously isolated compounds from these *Streptomyces* strains and found that macrotetrolide antibiotics such as dinactin and nactic acids (Al-Refai, 2008; Mahmoud, 2008), displayed motility inhibitory and lytic activities against zoospores which were identical to the effects in crude extracts. Dinactin is a member of the macrotetrolide complex produced by a range of *Streptomyces* species, which also includes nonactin, monactin, trinactin and tetranactin (Beck et al., 1962; Al-Refai, 2008; Mahmoud, 2008). These nactins are known to enhance ATPase activity in the mitochondria and cause rapid hydrolysis of ATP. They were also shown to act as monovalent cation ionophores with high selectivity for ammonium and potassium (Graven et al., 1966, 1967). The novel biological activities of macrotetrolides found in this study prompted us to further test structurally related compounds of dinactin and some known ionophores to understand the structure-activity relationships as well as to get information on the mode of action of these natural products. Therefore, the objectives of our study were (i) to screen extracts of marine *Streptomyces* spp. on motility and viability of zoospores of *P. viticola*; (ii) to test compounds previously isolated from extracts of *Streptomyces* spp. that exhibited motility inhibitory and lytic activities against zoospores; (iii) to test compounds structurally related to (+)-nonactic acid and dinactin on motility and lysis of zoospores of *P. viticola, Phytophthora capsici* and *Aphanomyces cochlioides*; (iv) to evaluate the effect of further known compounds such as nigericin and gramicidin having similar mode of action on zoospores; and (v) to evaluate the effects of zoospore motility inhibitors on zoosporogenesis of *P. viticola*.

## Materials and methods

### Materials and experimental procedure

Macrotetrolide antibiotics such as monactin and trinactin and further ionophoric compounds, nigericin and gramicidin were purchased from Tebu-bio and Sigma-Aldrich, respectively. Dinactin, nonactin, bonactin, feigrisolide, (+)-nonactic acid, (+)-homonactic acid, nonactic acid methyl ester, and homonactic acid methyl ester available in the laboratory were either previously isolated from marine *Streptomyces* spp. (Act 8970 and ACT 7619) or synthesized. All other chemicals were of at least reagent grade. Stock solutions of test compounds were prepared in small amounts of dimethyl sulfoxide (DMSO) and then diluted with water. The concentration of DMSO in the incubation medium never exceeded 1%, a condition that does not affect motility and viability of peronosporomycete zoospore (Islam et al., 2011).

### Cultivation of marine *Streptomyces* spp. and extraction

The marine *Streptomyces* spp. strains (such as Act8970) used in this research were obtained from the collection of the Institute of Organic and Biomolecular Chemistry, University of Göttingen, Germany. These strains were pre-cultivated on ${\text{M}}_{2}^{+}$ medium (+ 50% sea water) agar plates at 28°C for 3 days. To upscale these strains, pieces of well colonized agar were added to 1 l shaker cultures. Each strain was propagated in 5 1l-Erlenmyer flasks each containing 200 ml of M_2_+ (50% sea water) for 11 days at 28°C on a linear shaker with 110 rpm. After extraction of water phase and cell mass with ethyl acetate, the obtained extract was subjected to the bioassay.

### Peronosporomycete strains, production of zoospores and bioassay

Sporangia of *P. viticola* were isolated from infected leaves of grapevine (*Vitis vinifera* cv. Müller-Thurgau) (Islam et al., 2011). This strain was originally gained from infected leaf materials of the grapevine cv. Riesling in 1996 and since then maintained and propagated on fresh leaves of cv. Müller-Thurgau kept on Petri dishes containing 1.5% agar at 25°C and 95% relative humidity (Islam and von Tiedemann, 2008, 2011). At day 6 of cultivation, the sporangiophores bearing lemon-shaped sporangia were harvested into an Eppendorf vial by a micro-vacuum cleaner. The freshly harvested sporangia were separated from sporangiophores by filtration through a nylon sieve (50 μm mesh), washed twice with distilled water and then incubated in sterilized tap water (3 × 10^4^ sporangia/mL) in the dark for 6 h at room temperature (23°C) to release zoospores. These zoospores remained motile for 10–12 h in sterilized water and were used for the bioassay (Islam et al., 2011). The bioassay for testing the effects of pure compounds on release of zoospores from sporangia was carried out as described earlier (Islam et al., 2011).

The bioassay on motility and lysis of zoospores in presence of varying doses of pure compounds was carried out as described earlier (Islam et al., 2002a, 2011). Briefly, 40 μL of sample solution was directly added to 360 μL of zoospore suspension (*ca*. 10^5^/mL) taken in a dish of a plant tissue culture multi-well plate to make a final volume of 400 μL and then quickly mixed with a glass rod; 1% aqueous DMSO was used as a control. The motility of zoospores was observed under a light microscope at 100-fold magnification. Quantification of time-course changes of motility and lysis of zoospores were carried out as described earlier (Islam et al., 2004). Each treatment was replicated five times. The mean value (%) ± SE (standard error) of the affected spores in each treatment was calculated.

### Cultivation of *Aphanomyces cochlioides* and *Phytophthora capsici*, production of zoospores and bioassay

The damping-off pathogen of sugar beet and spinach, *Aphanomyces cochlioides*, was obtained from the Sugar Beet Research Institute (IFZ) in Goettingen, Germany. The culture of this strain and protocol for production of zoospores are described elsewhere (Islam and von Tiedemann, 2011; Zohara et al., 2016). *P. capsici* was provided by Prof. W. Yuancaho of Nanjing Agricultural University, China, which was isolated from soil of Nanjing, China. This organism was cultured on V8 juice agar. Production of zoospores and bioassays were carried out following protocols reported earlier (Tareq et al., 2014; Zohara et al., 2016). Each treatment was replicated five times. The mean value (%) ± SE (standard error) of the affected spores in each treatment was calculated.

### Statistical analysis, experimental design/replications

Experiments for evaluating biological activities of the pure compounds were carried out using a complete randomized design (CRD). Data were analyzed by one way analysis of variance (ANOVA) and the mean values were separated by Tukey's HSD (honest significant difference) posthoc statistic. All the analyses were performed using SPSS (IBM SPSS statistics 21, Georgia, USA). Mean value ± standard error of 5 replications were used in Tables and Figures.

## Results

### Motility inhibitory and lytic activities of extracts of marine *Streptomyces* spp. strains

To see whether marine *Streptomyces* spp. produce inhibitory substances against notorious phytopathogenic peronosporomycetes, we tested crude extracts of a large number of previously studied strains on motility behavior of *P. viticola* zoospores that produce diverse bioactive secondary metabolites. Out of 89 crude extracts from different strains of marine *Streptomyces* spp. tested, strains Act 8970, B6167, B7857, ACT7619, and Gt-2005/009 displayed significantly higher (*p* ≤ 0.001) motility inhibitory and subsequent lytic activities against *P. viticola* zoospores at 10 μg/ml or lower concentrations (Table 1). The antibiotic activity of the crude extracts of strains Act 8970, B6167, B7857, Act7619, and Gt2005/2009 was due to the presence of macrotetrolide antibiotics as dinactin and nactic acids displayed identical motility inhibitory and lytic activities against the zoospores in a dose- and time-dependant manners. These compounds were isolated from all these strains in our laboratory (Al-Refai, 2008; Mahmoud, 2008; Rahman, 2008). Therefore, homologs of dinactin and nactic acids were used in further detailed inhibition bioassays toward zoosporogenesis and motility of zoospores of *P. viticola*. To see whether the inhibitory activities of macrotetrolides and nactic acids are specific to *P. viticola* or general to other economically important phytopathogenic peronosporomycetes, we included a damping-off pathogen of sugar beet and spinach, *Aphanomyces cochlioides* and a late blight pathogen of chili and several vegetables, *Phytophthora capsici*.

**Table 1:** **Motility halting and zoosporicidal activity of marine ***Streptomyces*** spp. extracts against the downy mildew pathogen ***Plasmopara viticola*****.

**Name of extract**	**Dose (μg/ml)**	**Motility halting and zoosporicidal activity (% ± SE)^†^**							
		**15 min**		**30 min**		**45 min**		**60 min**	
		**Halted**	**Burst**	**Halted**	**Burst**	**Halted**	**Burst**	**Halted**	**Burst**
Act 8970	1	0±0	0±0	0±0	0±0	0±0	0±0	11±1.2	0±0
	5	3±0.6	0±0	7±2.3	0±0	17±2.9	5±2.3	48±3.5	36±2.3
	10	62±4.6	29±2.3	79±3.5	58±4.0	86±3.5	75±5.2	98±1.2	84±4.0
B6167	1	0±0	0±0	0±0	0±0	0±0	0±0	0±0	0±0
	5	0±0	0±0	5±1.7	0±0	22±2.9	9±1.2	43±4.0	15±4.6
	10	47±3.5	23±2.9	68±2.3	37±5.2	86±2.3	69±2.9	88±1.2	77±3.5
B7857	1	0±0	0±0	0±0	0±0	0±0	0±0	0±0	0±0
	5	0±0	0±0	9±1.2	0±0	23±2.3	18±1.7	39±3.5	21±2.3
	10	49±2.9	11±4.0	62±4.6	28±5.2	75±4.0	41±4.6	87±1.8	69±5.2
Act7619	1	0±0	0±0	0±0	0±0	0±0	0±0	0±0	0±0
	5	0±0	0±0	7±1.7	0±0	22±2.9	9±1.2	43±4.0	30±4.0
	10	47±2.3	13±2.9	58±2.3	32±5.2	78±5.8	49±2.9	88±1.2	71±3.5
Gt-2005/009	1	0±0	0±0	0±0	0±0	0±0	0±0	0±0	0±0
	5	0±0	0±0	7±2.3	0±0	24±3.5	16±1.7	40±4.6	22±2.3
	10	57±4.0	9±1.7	68±5.2	36±5.2	79±5.8	59±4.0	90±3.5	67±4.6
B5136	1	20±1.2	0±0	28±2.9	0±0	38±1.7	0±0	54±4.0	0±0
	5	99±0.6	9±0.6	100±0	88±4.0	100±0	100±0	100±0	100±0
	10	100±0	85±3.5	100±0	100±0	100±0	100±0	100±0	100±0
B3497	1	0±0	0±0	0±0	0±0	14±1.2	0±0	38±1.7	18±2.9
	5	96±1.7	81±3.5	99±0.6	88±2.9	100±0	92±2.9	100±0	100±0
	10	99±0.6	85±4.0	100±0	93±2.9	100±0	100±0	100±0	100±0
B4818	1	0±0	0±0	0±0	0±0	0±0	0±0	0±0	0±0
	5	78±3.5	52±3.5	100±0	78±2.9	100±0	95±2.3	100±0	100±0
	10	98±1.2	98±1.2	100±0	100±0	100±0	100±0	100±0	100±0
B7798	1	0±0	0±0	0±0	0±0	0±0	0±0	0±0	0±0
	5	78±3.5	45±2.3	88±4.6	60±3.5	95±2.9	73±4.0	100±0	100±0
	10	98±1.2	75±4.6	100±0	100±0	100±0	100±0	100±0	100±0
B7060	5	0±0^*^	0±0	0±0^*^	0±0	45±2.3	26±1.7	93±2.9	76±3.6
	10	98±1.2	0±0	100±0	55±3.5	100±0	78±4.6	100±0	82±5.2
	50	100±0	72±4.0	100±0	81±5.2	100±0	86±5.2	100±0	92±3.5
B8774	10	0±0	0±0	0±0	0±0	0±0	0±0	0±0	0±0
	50	99±0.6	65±5.2	100±0	75±3.5	100±0	82±4.6	100±0	99±0.6
	100	100±0	80±4.6	100±0	95±2.3	100±0	100±0	100±0	100±0
B7747	10	0±0	0±0	0±0	0±0	0±0	0±0	0±0	0±0
	50	98±1.2	35±4.6	100±0	42±2.9	100±0	60±3.5	100±0	69±3.5
	100	100±0	56±2.9	100±0	72±4.0	100±0	80±5.2	100±0	88±4.6
B4842	10	0±0	0±0	18±1.7	9±0.6	28±1.2	12±2.3	35±2.9	26±1.2
	50	100±0	10±1.2	100±0	28±2.9	100±0	43±2.9	100±0	64±3.5
	100	100±0	41±3.5	100±0	52±3.5	100±0	72±5.2	100±0	80±5.2
B5530	10	0±0	0±0	35±2.3	0±0	39±3.5	0±0	46±2.3	0±0
	50	100±0	45±3.5	100±0	63±3.5	100±0	78±5.8	100±0	80±3.5
	100	100±0	70±4.6	100±0	83±4.0	100±0	95±2.9	100±0	100±0
B4677	10	0±0	0±0	0±0	0±0	6±0.6	0±0	10±1.2	0±0
	50	100±0	48±4.6	100±0	80±5.2	100±0	88±3.5	100±0	91±4.0
	100	100±0	98±1.2	100±0	100±0	100±0	100±0	100±0	100±0
B8300	50	60±4.0	0±0	72±2.9	0±0	88±4.6	0±0	100±0	0±0
	100	100±0	0±0	100±0	23±2.3	100±0	36±3.5	100±0	49±0
B8690	50	94±2.3	0±0	92±1.2	47±2.9	93±2.9	67±4.6	100±0	80±5.2
	100	100±0	40±3.5	100±0	69±4.6	100±0	83±3.5	100±0	100±0
B8251	50	79±2.9	0±0	88±1.2	30±4.0	98±1.2	42±3.5	100±0	55±2.3
	100	100±0	39±4.6	0±0	59±2.3	100±0	63±2.3	100±0	70±5.2
B9042	10	26±2.3	0±0	48±1.2	20±2.3	65±4.6	32±2.9	82±5.2	72±3.5
	50	67±4.6	49±4.0	87±2.3	62±1.7	94±2.3	79±4.0	100±0	93±2.3
B7936	10	0±0	0±0	18±2.3	6±0.6	49±4.0	25±2.8	72±4.0	51±4.0
	50	57±3.5	31±4.0	77±4.6	40±3.5	86±5.2	56±3.5	94±3.5	83±2.9
B8160	10	18±1.7	0±0	29±3.5	12±0.6	52±3.5	24±2.3	88±3.5	43±2.3
	50	67±2.3	41±3.5	89±2.9	57±5.1	92±2.3	72±4.0	100±0	94±2.9
B1638a	10	38±2.9	7±1.7	47±4.0	19±2.3	68±4.0	33±3.5	85±4.0	46±3.5
	50	76±4.6	53±2.9	93±3.5	68±4.0	100±0	88±2.9	100±0.0	90±2.9
B4854	10	5±0.6	0±0	19±2.3	0±0	34±2.3	0±0	51±2.9	14±1.2
	50	81±3.5	43±3.5	92±4.0	67±4.0	100±0	95±2.3	100±0	97±1.7
	100	99±0.6	90±2.9	100±0	95±2.3	100±0	100±0	100±0	100±0

† *One way ANOVA was performed and data in column varies significantly at p ≤ 0.001. Post-hoc tests could not be performed because the number groups is more than 50*.

* *Zoospores (100%) moved to bottom and move very slowly; We selected strains those previously found to produce antibiotics against pathogenic microorganisms*.

### Motility inhibitory and lytic activities of macrotetrolide antibiotics and nactic acids against *P. viticola* zoospores

**Figure 1:**
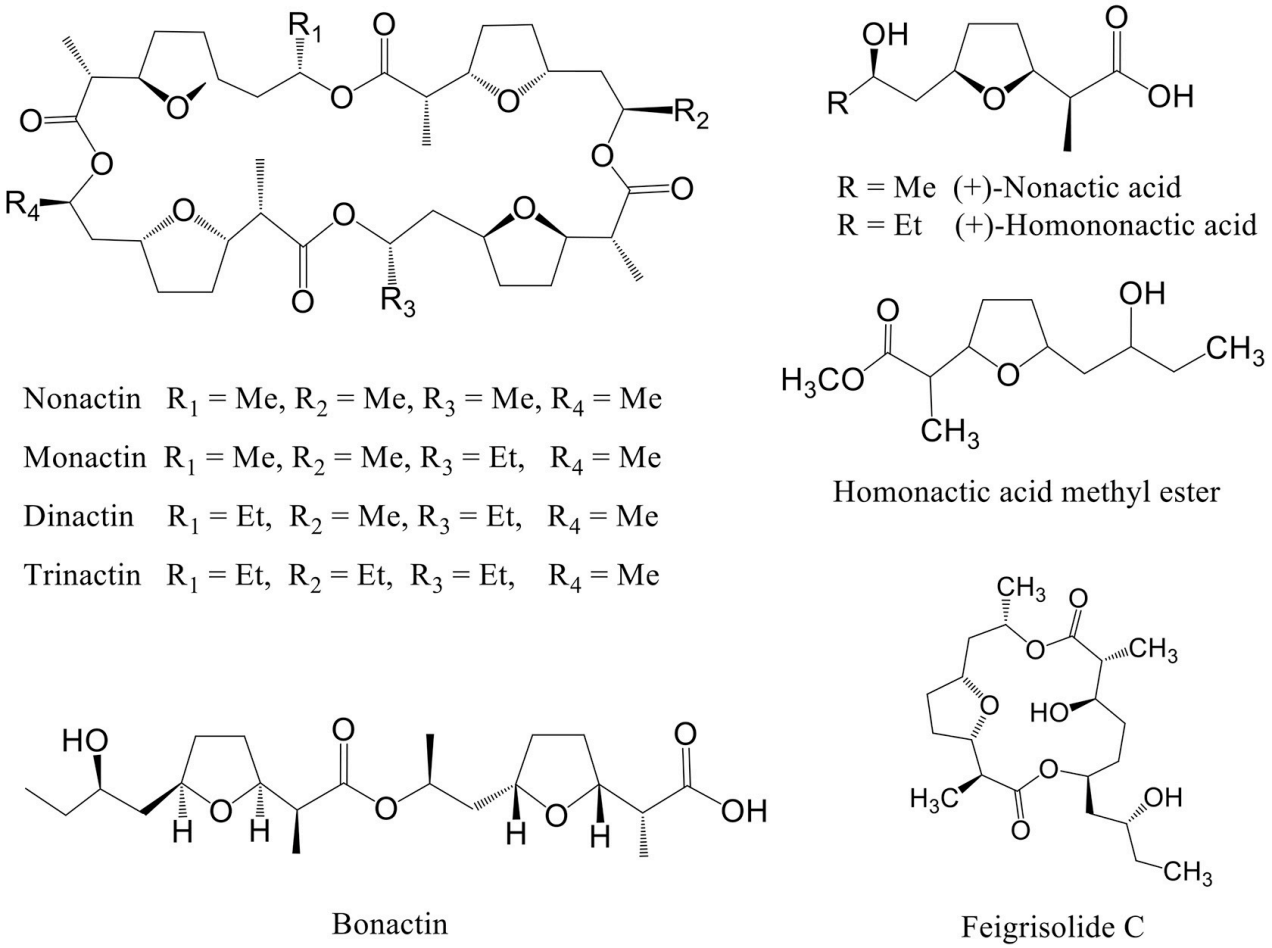
**Structure of secondary metabolites isolated from marine ***Streptomyc***es spp. having motility inhibitory and lytic activities against peronosporomycete zoospores**.

**Figure 2:**
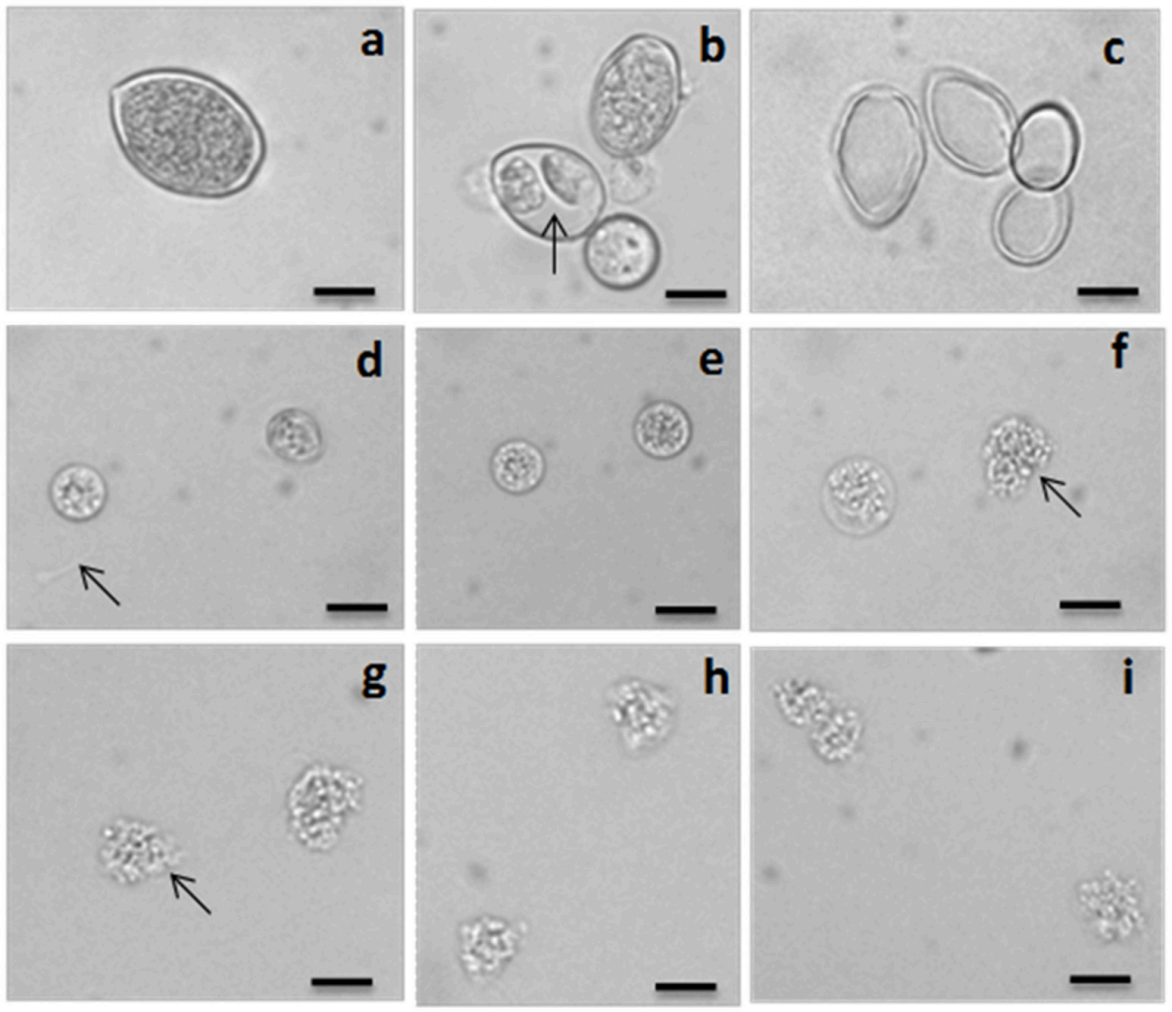
**Light micrographs showing sporangium, differentiating sporangium, empty sporangium (ghost) and inhibitory effects of dinactin, trinactin and gramicidin on zoospores of the grapevine downy mildew pathogen ***Plasmopara viticola***. (a)** A mature freshly harvested sporangium, **(b)** differentiated sporangia with developed zoospores (arrow) inside, **(c)** empty sporangia (ghosts) after release of zoospores, **(d)** two halted zoospores at the bottom of the dish just after addition of dinactin (1 μg/ml). Arrow indicates the trace of a halted zoospore, **(e)** both halted zoospores become round by 5 min after treatment with dinactin (1 μg/ml), **(f)** disruption of membrane and granulation of cell organelle and lysis (arrow) of zoospores by dinactin 10 min after treatment (1 μg/ml), **(g)** lysis of zoospores by dinactin 15 min after treatment (1 μg/ml), **(h)** lysis of zoospores by trinactin 15 min after treatment (1 μg/ml), **(i)** lysis of zoospores by gramicidin 15 min after treatment (1 μg/ml). Bar represents 10 μm.

Compounds previously isolated from strains of Act 8970 viz. dinactin, (+)-nonactic acid, (+)-homononactic acid, homononactic acid methyl ester, and nonactic acid methyl ester, were tested on motility of *P. viticola* zoospores (Table 2 and Figure 1). Among them, the macrotetrolide antibiotic dinactin displayed the highest potency in arresting motility and caused subsequent lysis of *P. viticola* zoospores starting from 0.3 μg/ml (Table 2). The activities of the linear nactic acids and their methyl esters were 5–50-fold lower in inhibition of zoospore motility than of dinactin. Dinactin inhibited the motility completely and caused lysis of all stopped zoospores (100%) within 15 min exposure to the compound at 1 μg/ml. One way ANOVA revealed that zoospore motility inhibitory activities of the varying concentrations of the tested compounds varied significantly at *p* ≤ 0.05. Microscopic observation revealed that in presence of dinactin, swimming of zoospores was rapidly impaired or slowed down and/or zoospores spun in tight circles for a short time (Figure 2). Finally, all affected zoospores stopped moving and most of them subsequently lysed within several minutes of treatment, depending on the concentration of the compound. Before lysis, the cellular materials in halted zoospores rapidly became granulated and then gradually fragmented and dispersed into the surrounding water upon burst of their cell membranes (Figure 2f). In contrast, *P. viticola* zoospores in untreated control dishes exhibited the characteristic helical swimming almost following a straight line for several hours. Other nactic acids and their methyl esters also impaired motility of zoospores and caused subsequent lysis in an identical manner but at varying concentrations (Table 2). Among them, (+)-homonactic acid exhibited the strongest activity in both inhibition of motility and lysis of zoospores followed by nonactic acid methyl ester, homononactic methyl ester and (+)-nonactic acid in decreasing order. (+)-Homononactic acid inhibited motility of 100% zoospores at 5 μg/ml by 60 min of treatment, which was 10-fold stronger activity compared to the activity of (+)-nonactic acid (Table 2). The zoospore lytic activities of the tested compounds also varied significantly at *p* ≤ 0.05.

**Table 2:** **Motility halting and zoosporicidal activity of nactic acids and their esters and dinactin isolated from marine ***Streptomyces*** sp. Act 8970 against the grapevine downy mildew pathogen ***Plasmopara viticola*****.

**Compound**	**Dose (μg/ml)**	Motility halting and zoosporicidal activity (% ± SE)^*^							
		**15 min**		**30 min**		**45 min**		**60 min**	
		**Halted**	**Burst**	**Halted**	**Burst**	**Halted**	**Burst**	**Halted**	**Burst**
(+)-Nonactic acid	10	0±0f	0±0f	0±0e	0±0g	0±0e	0±0f	0±0e	0±0g
	30	71±4c	56±2c	80±2bc	72±4c	82±3c	75±4bc	88±4b	82±4de
	50	90±4b	82±5b	98±1a	86±4b	100±0a	95±2a	100±0a	98±2ab
	100	100±0a	100±0a	100±0a	100±0a	100±0a	100±0a	100±0a	100±0a
(+)-Homonactic acid	1	0±0f	0±0f	0±0e	0±0g	0±0e	0±0f	0±0e	0±0
	5	91±2ab	88±4b	97±2a	90±2ab	99±1a	95±2a	100±0a	98±1ab
	10	100±0a	100±0a	100±0a	100±0a	100±0a	100±0a	100±0a	100±0a
Nonactic acid methyl ester	5	0±0f	0±0f	0±0e	0±0g	0±0e	0±0f	0±0e	0±0g
	10	35±4e	19±1e	71±4c	46±4de	79±3c	70±5c	89±4b	83±4de
	20	52±3d	32±3d	79±4bc	54±3d	85±3bc	73±6bc	100±0a	88±5bd
Homonactic methyl ester	10	0±0f	0±0f	0±0e	0±0g	0±0e	0±0f	0±0a	0±0g
	20	0±0f	0±0f	0±0e	0±0g	10±1e	0±0f	25±2d	0±0g
	30	60±1d	30±2d	72±3c	42±5e	88±6bc	59±3d	100±0a	75±2e
Dinactin	0.1	0±0f	0±0f	0±0e	0±0g	0±0e	0±0f	0±0e	0±0g
	0.3	0±0f	0±0f	31±2d	19±2f	40±3d	23±6e	53±2c	42±4f
	0.5	75±2c	63±4c	83±3b	74±4c	92±4ab	82±5b	96±2ab	93±3ac
	1.0	100±0a	100±0a	100±0a	100±0a	100±0a	100±0a	100±0a	100±0a

* *Means within a column followed by the same letter(s) are not significantly different as assessed by Tukey's HSD (honest significance difference) post-hoc (p ≤ 0.05)*.

### Biological activity of compounds structurally related to dinactin and dinactic acids

Dinactin is a member of the macrotetrolide complex produced by a range of *Streptomyces* species which includes several homologs such as nonactin, monactin, and trinactin (Beck et al., 1962). Early literature reported that these compounds almost equally enhance mitochondrial ATPase activity and cause rapid hydrolysis of ATP (Graven et al., 1966). They are also known to act as monovalent cation ionophore with high selectivity for ammonium and potassium (Graven et al., 1967) and have diverse biological activities (Zizka, 1998). To get insight into the structure-activity relationships, we tested some homologs of dinactin such as nonactin, monactin and trinactin together with two linear compounds such as bonactin and feigrisolide C previously isolated from marine *Streptomyces* species (Figure 1).

**Figure 3:**
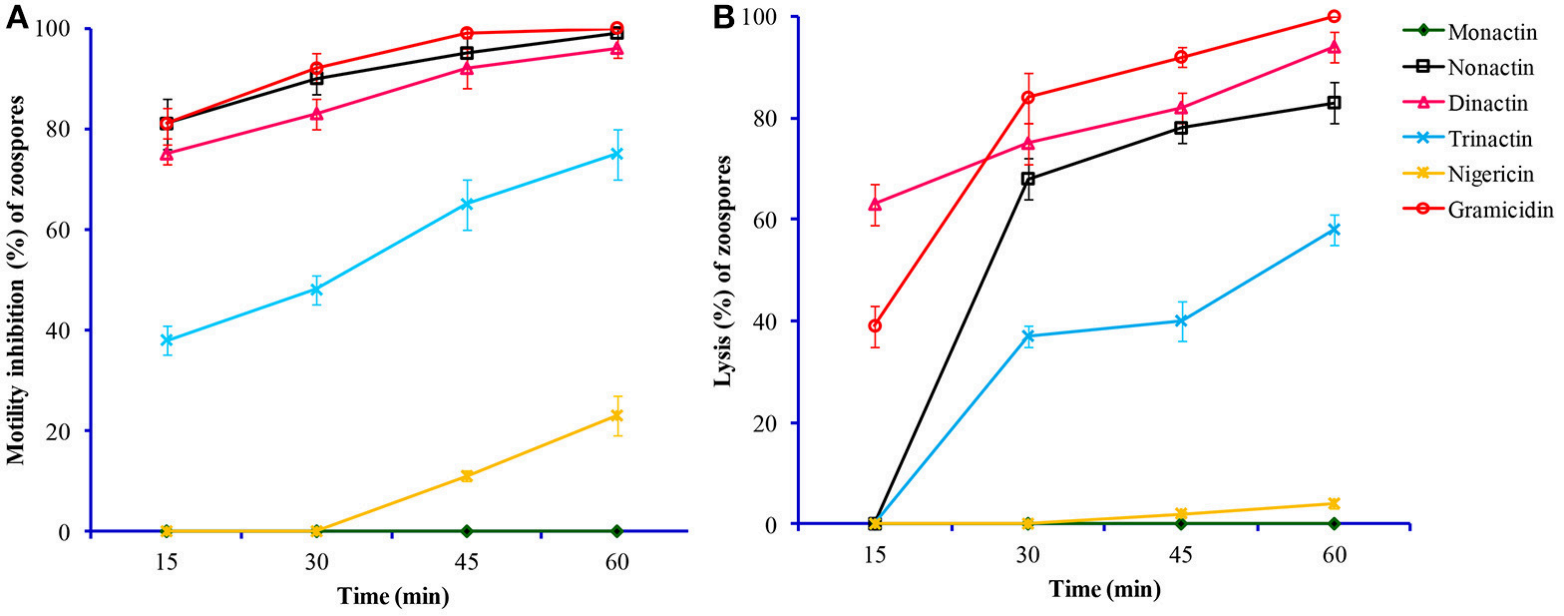
**Time-course comparative motility inhibitory (A)** and lytic activities **(B)** of macrotetrolide antibiotics against zoospores of the grapevine downy mildew pathogen *Plasmopara viticola* at 0.5 μg/ml (*p* ≤ 0.05).

All three homologs (nonactin, monactin, and trinactin) of dinactin displayed motility impairing and lytic activities against *P. viticola* zoospores in an identical fashion and dose- and time-dependent manner (Table 3). One way ANOVA revealed that zoospore motility inhibitory activities of the tested compounds and their different concentrations varied significantly at *p* ≤ 0.05. The strengths of activity of all these homologs were similar. Bonactin and feigrisolide C also exhibited motility inhibitory and lytic activities in a similar manner but required almost 10-fold higher doses compared to dinactin. Time-course investigation revealed that dinactin and trinactin caused 100% inhibition of zoospore motility within 15 min exposure to the tested compounds at 1 μg/ml (Figure 3A). At the same concentration, nonactin and monactin also inhibited motility by 100% but required longer time, i.e., 30 and 60 min, respectively. All compounds showed almost similar phenomena in causing lysis of halted zoospores (Figure 3B). Initially, zoospores became paralyzed or moved very slowly in tight circles, stopped and then rapidly immobilized (Figure 2). The zoospore lytic activities of the varying concentration of the tested compounds also varied significantly at *p* ≤ 0.05.

**Table 3:** **Motility inhibitory and zoosporicidal activity of bonactin, feigrisolide C, macrotetrolide antibiotics (nonactin, monactin, dinactin and trinactin), nigericin and gramicidin against the grapevine downy mildew pathogen ***Plasmopara viticola*****.

**Compound**	**Dose (μg/ml)**	**Motility halting and zoosporicidal activity (% ± SE)^†^**							
		**15 min**		**30 min**		**45 min**		**60 min**	
		**Halted**	**Burst**	**Halted**	**Burst**	**Halted**	**Burst**	**Halted**	**Burst**
Bonactin	1.0	0±0h	0±0g	43±4e	31±4g	58±2d	41±2e	71±4c	63±4d
	2.0	87±4bc	67±5d	92±3ac	88±2bc	96±3ab	93±4ab	99±1ab	97±3ab
	5.0	100±0a	85±5bc	100±0a	100±0a	100±0a	100±0a	100±0a	100±0a
	10.0	100±0a	100±0a	100±0a	100±0a	100±0a	100±0a	100±0a	100±0a
Feigrisolide C	1.0	0±0h	0±0g	0±0g	0±0i	8±1f	4±1g	24±4f	18±3f
	5.0	27±4g	0±0g	37±4ef	12±2h	43±2e	18±3f	51±5d	41±3e
	10.0	96±2ab	88±5ab	100±0a	100±0a	100±0a	100±0a	100±0a	100±0a
Nonactin	0.1^s^	0±0h	0±0g	0±0g	0±0i	12±1f	0±0g	32±2ef	0±0g
	0.5	81±5df	0±0g	90±3ac	68±4e	95±3ab	78±3cd	99±1ab	83±4c
	1.0	95±3ac	81±6bc	100±0a	97±2ab	100±0a	100±0a	100±0a	100±0a
Monactin	0.1^s^	0±0h	0±0g	0*b*±0g	0±0i	0±0f	0±0g	0±0g	0±0g
	0.5	0±0h	0±0g	0*b*±0g	0±0i	0±0f	0±0g	0±0g	0±0g
	1.0	88±4ad	73±4cd	96±1ab	92±2ac	99±1a	98±1a	100±0a	99±1a
	2.5	100±0a	100±0a	100±0a	100±0a	100±0a	100±0a	100±0a	100±0a
Dinactin	0.1^s^	0±0h	0±0g	0±0g	0±0i	0±0f	0±0g	0±0g	0±0g
	0.3	0±0h	0±0g	31±2f	19±2h	40±3e	23±3f	53±2d	42±4e
	0.5	75±2ef	63±4d	83±3bc	74±4de	92±4ac	82±3bd	96±2ab	93±3ac
	1.0	100±0a	100±0a	100±0a	100±0a	100±0a	100±0a	100±0a	100±0a
Trinactin	0.1^s^	0±0h	0±0g	7*c*±1g	0±0i	12±2f	3±1g	39±3e	28±3f
	0.5	38±3g	0±0g	48±3e	37±2g	65±5d	40±4e	75±5c	58±3d
	1.0	100±0a	100±0a	100*f*±0a	100±0a	100±0a	100±0a	100±0a	100±0a
	2.5	100±0a	100±0a	100*f*±0a	100±0a	100±0a	100±0a	100±0a	100±0a
Nigericin	0.5	0±0h	0±0g	0±0g	0±0i	11±1f	0±0g	23±4f	0±0g
	1.0d	32±4g	0±0g	63±3d	11±1hi	80±3c	72±5d	94±3ab	88±4bc
	2.5	72±5f	6±1fg	81±4c	18±2h	89±7ac	85±4bc	99±1ab	98±1ab
	5.0	82±2cf	38±3e	94±2ab	81±3cd	99±1a	89±4ac	100±0a	100±0a
Gramicidin	0.05	0*e*±0h	0±0g	0±0g	0±0i	0±0f	0±0g	0±0g	0±0g
	0.1	70±3f	15±1f	82±5c	51±4f	84±5bc	72±4d	88±3b	86±5c
	0.5	81±3df	39±4e	92±3ac	84±5cd	99±1a	92±2ab	100±0a	100±0a
	1.0	100±0a	100±0a	100*f*±0a	100±0a	100±0a	100±0a	100±0a	100±0a

† *Means within a column followed by the same letter(s) are not significantly different as assessed by Tukey's HSD (honest significance difference) post-hoc (p ≤ 0.05)*.

s *Stimulant and remained middle layer of water, spiral swimming*.

### Motility of zoospores in presence of gramicidin and nigericin

To better assess whether induction of ATPase activity and hydrolysis of ATP in mitochondria or any other mechanism is associated with motility inhibitory and lytic activities of zoospores by macrotetrolide antibiotics, a channel-forming ionophore, gramicidin and a mobile carrier of cations through plasma membranes, nigericin were tested (Graven et al., 1966, 1967). Gramicidin showed the highest activity (*p* ≤ 0.05) among the tested compounds, which was 2-fold stronger than those of macrotetrolides for inhibition of zoospore motility by 100% (Table 3). On the other hand, nigericin also displayed zoospore motility arresting activity but had 10-fold weaker efficacy compared with gramicidin. In both cases, halted zoospores were lysed in a similar manner as shown by the macrotetrolide antibiotics. On the other hand, nigericin displayed almost equal strength to bonactin in arresting motility of 100% zoospores at 5 μg/ml, while the dose required for equivalent efficacy by feigrisolide was 10 μg/ml (Table 3). Motility inhibitory and lytic activities against zoospores by gramicidin and nigericin were statistically significant (*p* ≤ 0.05).

### Effects of macrotetrolide antibiotics on motility of *Aphanomyces cochlioides* zoospores

To evaluate whether motility inhibitory and lytic activities of marotetrolides are common phenomena in Peronosporomycete zoospores, we tested all homologs of dinactin and other bioactive compounds evaluated on *P. viticola* against a sugar beet damping-off pathogen *Aphanomyces cochlioides* (Peronosporomycete). An almost identical phenomenon was observed when motile *A. cochlioides* zoospores were exposed to macrotetrolide antibiotics or other inhibitors, but surprisingly none of the compounds caused any lysis of the halted zoospores until 60 min (Table 4). One way ANOVA revealed that zoospore motility inhibitory and lytic activities of the tested compounds and their varying concentrations varied significantly at *p* ≤ 0.001. Irrespective of the test compounds, all motility-impaired zoospores rapidly became round cystospores instead of lysis, however, none of them germinated until 60 min after the treatment (data not shown).

**Table 4:** **Motility inhibitory activity of nactic acids and their esters, and dinactin isolated from marine ***Streptomyces*** sp. Act 8970 against the sugar beet damping-off pathogen ***Aphanomyces cochlioides*** AC-1**.

**Compound**	**Dose (μg/ml)**	Motility inhibitory activity^*^ (60 min) (% ± SE)
(+)-Nonactic acid	10	0±0d
	20	37±3c
	40	88±5b
	80	95±2ab
(+)-Homonactic acid	1	0±0d
	5	98±1ab
	10	100±0a
Nonactic acid methyl ester	5	0±0d
	10	89±5ab
	20	100±0a
Homonactic methyl ester	10	0±0d
	20	88±5ab
	30	100±0a
Dinactin	0.1	0±0d
	0.5	98±1ab
	1.0	100±0a

* *Data are average values ± SE of of five replications in each dose of each tested compound. Dinactic acid was inactive up to 100 μg/ml. No lysis of zoospores was observed until 60 min of treatment by any of the tested compounds. Data were analyzed by one way ANOVA [F1_(5, 32)_ = 417.3, p < 0.001]. Mean values were separated by Tukey's HSD (honest significance difference) posthoc statistic*.

### Motility inhibitory and lytic activities of dinactin and trinactin against *Phytophthora capsici* zoospores

**Figure 4:**
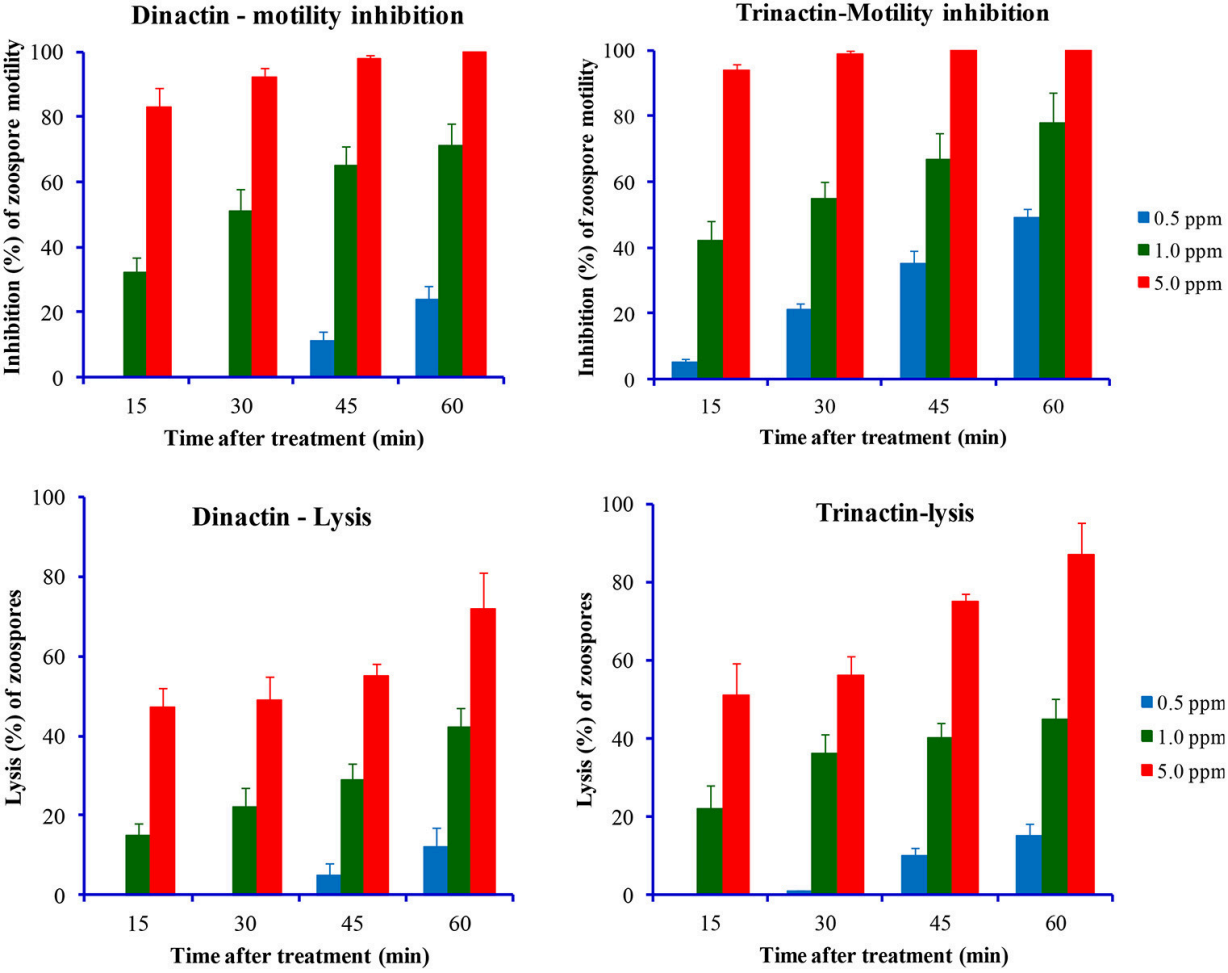
**Time-course comparative motility inhibitory (upper panel) and lytic activities (lower panel) of dinactin and trinactin against zoospores of the late blight pathogen of chili and cucumber, ***Phytophthora capsici*** at varying doses of tested compounds**.

Two homologs of macroterolide antibiotics, dinactin and trinactin were also tested against the zoospores of another notorious peronosporomycete phytopathogen, *Phytophthora capsici*. As expected, both compounds displayed inhibitory activity against *P. capsici* zoospores in a dose- and time-dependant manner. Trinactin showed significantly (*p* ≤ 0.001) stronger inhibitory activity compared with dinactin (Figure 4). The zoospores of *P. capsici* seemed less sensitive to the macrotetrolides compared to *P. viticola* zoospores. The IC_50_ values for motility inhibition of zoospores by dinactin and trinactin were ca. 1.0 and 0.5 μg/ml, respectively. Unlike *P. viticola* zoospores, only a small fraction of motility impaired zoospores became lysed by the treatment of dinactin and trinactin. Approximately, 50% of the halted zoospores became round cystospores and failed to germinate until 60 min after the treatments.

### Inhibition of zoosporogenesis by nonactic acid and macroterolides

**Figure 5:**
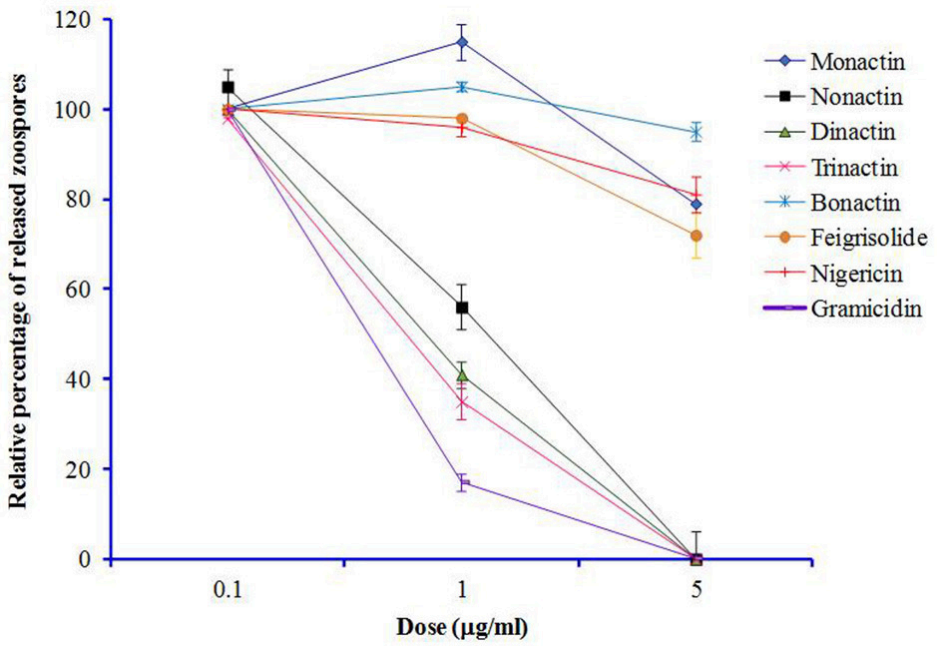
**Effects of macrotetrolide antibiotics, and bonactin, feigrisolide C, nigericin and gramicidin on relative percentage (%) of release of zoospores from sporangia (zoosporogenesis) of grapevine downy mildew pathogen ***Plasmopara viticola*** over untreated control (***p*** ≤ 0.001)**.

Freshly harvested and washed *P. viticola* sporangia (3 × 10^5^/ml) typically release zoospores up to 1 × 10^6^/ml in sterilized water within 5–6 h. We tested whether macrotetrolide antibiotics (nonactin, monactin, dinactin, and trinactin), bonactin, feigrisolide, nigericin and gramicidin, have an effect on the process of zoospore release (i.e., zoosporogenesis). The bioassay revealed that all compounds significantly (*p* ≤ 0.001) inhibited zoosporogenesis in a dose-dependent manner but in varying concentrations (Table 5 and Figure 5). Zoosporogenesis was completely blocked by monactin, dinactin, trinactin and gramicidin at 5 μg/ml. At lower doses of these compounds, the release of zoospores still occurred but most zoospores became immobilized soon after release. Nonactin also displayed a similar inhibitory effect but required a 2-fold higher concentration for equivalent activity to other macrotetrolides. The bonactin and feigrisolide also suppressed the release of zoospores but required several fold higher concentrations compared with dinactin. Nigericin had weak activity (IC_50_ 10 μg/ml) in suppressing zoosporogenesis of *P. viticola*.

**Table 5:** **Effects of macrotetrolide antibiotics, and bonactin, feigrisolide C, nigericin and gramicidin on the release of zoospores from sporangia (zoosporogenesis) of the grapevine downy mildew pathogen ***Plasmopara viticola*****.

**Compound**	**Dose(μg/ml)**	Relative percent of released zoospores (% ± SE) and their motility behavior^*^	
		**Zoospores**	**Behaviors or fate of released zoospores**
Nonactin	0.1	105±4ac	Normal swimming
	0.5	120±6a	Normal swim ming
	1.0	115±5ab	Swam faster than normal speed
	5.0	79±6fg	No motile zoospores and 100% zoospores lysed just after release
	10.0	0±0l	–
Monactin	0.1	100±0bd	Normal swimming
	1.0	56±4hi	80% of the released zoospores lysed and others swam straight with less or no turning. Speed of swimming was normal.
	5.0	0±0l	–
Dinactin	0.1	100±0bd	Normal swimming
	1.0	41±3ij	50% of released zoospores lysed and others swam normally
	5.0	0±0l	–
Trinactin	0.1	98±1cd	Normal swimming
	1.0	35±4j	Presence of debris at the bottom of dish, all released zoospores lysed.
	5.0	0±0l	–
Bonactin	0.1	100±1bd	Normal swimming
	1.0	105±1ac	Normal swimming
	5.0	95±2cf	Normal swimming
	10.0	85±5dg	95% of released zoospores lysed and 5% had normal swimming
Feigrisolide C	0.1	100±0bd	Normal swimming
	1.0	98±1cd	95% normal swimming and 5% lysed.
	5.0	72±5gh	80% of released zoospores lysed, others normal swimming
	10.0	7±1kl	100% of released zoospores lysed
Nigericin	0.1	100±0bd	Normal swimming
	1.0	96±2cd	Normal swimming
	5.0	81±4eg	Normal swimming
	10.0	47±5ij	90% of released zoospores lysed. Those moving were abnormal in size (bigger) and irregular in shape. No normal zoospores.
Gramicidin	0.1	100±0bd	Normal swimming
	0.5	75±4g	75% released zoospores lysed and others slowly swimming
	1.0	17±2k	100% released zoospores lysed
	5.0	0±0l	–

* *Data presented here are average values ± SE of five replications in each dose of compounds. Data were analyzed by one way ANOVA [F1_(26, 60)_ = 2009, p < 0.001]. Mean values were separated by Tukey's HSD (honest significance difference) posthoc statistic*.

## Discussion

In this study, we demonstrated that macrotetrolide antibiotics such as dinactin and nactic acids isolated from marine *Streptomyces* spp. impaired motility and caused lysis of *P. viticola* zoospores that are key stages of this devastating pathogen of grapevine (Tables 2, 3). These bioactive compounds also inhibited motility of *P. capsici* and *A. cochlioides* zoospores in a similar way (Table 4) and suppressed the release of zoospores from *P. viticola* sporangia in a dose-dependent manner (Table 5). In addition, the homologs of dinactin (e.g., nonactin, monactin, trinactin), which are known as enhancers of mitochondrial ATPase activity similar to nigericin and gramicidin also suppressed zoosporogenesis, impaired motility and caused lysis of *P. viticola* zoospores (Table 5, Figures 3, 4). Taken together, our results show for the first time that macroterolide antibiotics and nactic acids from marine *Streptomyces* spp. and other bacteria suppress zoosporogenesis and impair motility of peronosporomycete zoospores. Furthermore, preliminary bioassay revealed that one of the macrotetrolide, dinactin suppressed the sporulation of *P. viticola* in artificially inoculated grapevine leaf disks (data not shown) indicating its potential as a natural peronosporomicide. A further *in vivo* study is warranted to test this hypothesis. As zoosporogenesis and motility of zoospores are high energy demanding processes, the mode of action of these inhibitory activities by macroterolides is likely linked with the hydrolysis of mitochondrial ATP through enhanced ATPase activity. Motility inhibition and subsequent lysis of Peronosporomycete zoospores by various kinds of natural products such as indolocarbazole alkaloid, staurosporine (Islam et al., 2011), khatmiamycin from *Streptomyces* sp. ANK313 (Abdalla et al., 2011), isocoumarins from *Streptomyces* sp. ANK302 (Zinad et al., 2011), macrocyclic lactam antibiotics from *Lysobacter* sp. SB-K88 (Islam et al., 2005), polyflavonoid tannins from the bark of *Lannea coromandelica* (Islam et al., 2002a), anacardic acids from *Ginkgo biloba* (Begum et al., 2002), and polyketides and depsidones from the fungal endophyte *Cryptosporiopsis* sp. CAFT122-2 and *Phomopsis* sp. CAFT69, respectively (Talontsi et al., 2012a,b) have been reported previously. Impairment of motility and lysis of peronosporomycete zoospores by oligomycins and pamamycin homologs from marine *Streptomyces* have also been reported (Dame et al., 2016)

A hallmark of our finding is that all four macrotetrolide antibiotics (nonactin, monactin, dinactin, and trinactin) produced by marine *Streptomyces* spp. displayed qualitatively and quantitatively similar motility-impairing activities against the phytopathogenic peronosporomycete zoospores (Table 3 and Figures 3, 4). Furthermore, an ion-forming peptide gramicidin also displayed strong motility-impairing effects against the zoospores. As zoospores are unable to take up nutrients from their environment for maintenance of motility, they require steady supply of energy (ATP) from the internal cellular energy reserves (β-1,3-glucan or mycolaminarins) (Bimpong, 1975). The ATPase activity per volume of zoospores is similar to that of contracting skeletal muscles (Holker et al., 1993; Stienen et al., 1996). Therefore, disruption of energy supply from mitochondria causes impairment of swimming behavior of the zoospores. Both macrotetrolide antibiotics and gramicidin have been found to enhance ATPase activity and cause rapid hydrolysis of ATP in the mitochondria (Graven et al., 1966). Therefore, the motility inhibitory effect of same macrotetrolide antibiotics and gramicidin shown in this study is likely to be linked to the depletion of ATP by enhanced ATPase activity in the mitochondria of treated zoospores. The underlying molecular mechanism of maintenance of zoospore motility is still poorly understood. As antibiotics have been used as effective tools for many metabolic studies, a further quantitative study on ATPase activity in the mitochondria of zoospores treated with varying doses of macrotetrolide antibiotics should shed light on motility pathway of zoospores and may be used in the search for new targets for controlling this notorious class of phytopathogens. Inhibition of zoospore motility through disruption of cytoskeletal filamentous actin by microbial metabolites such as latrunculin B, 2,4-diacetylphloroglucinol and macrolyclic lactam antibiotic, xanthobaccin A has been reported (Islam, 2008; Islam and von Tiedemann, 2011).

Another novel finding of this study was suppression of zoospore release from sporangia of *P. viticola* by macrotetrolide antibiotics (Table 5 and Figure 5). The cation channel-former and inducer of mitochondrial ATPase activity, gramicidin also suppressed zoosporogenesis with very high efficacy. Although, the underlying molecular mechanisms of zoosporogenesis are still poorly understood (Judelson and Blanco, 2005), cleavage of nuclei and differentiation of sporangia during zoosporogenesis require supply of energy from mitochondria. Moreover, intracellular Ca^2+^ ions play important roles in zoosporogenesis (Islam and Tahara, 2001). Therefore, suppression of zoosporogenesis by known inducers (macrotetrolides and gramicidin) of mitochondrial ATPase activity and ionophores suggests that depletion of ATP in mitochondria by hydrolysis of ATP in concert with translocation (efflux/influx) of cations from the cells might be involved in this process. Suppression of zoosporogenesis in *P. viticola* by staurosporine from a marine *Streptomyces* sp. B5136 (Islam et al., 2011) and 2,4-diacetylphloroglucinol from the soil bacterium *Pseudomonas fluorescens* have previously been reported (Islam and von Tiedemann, 2011). Moreover, a selective inhibitor of protein kinase C (PKC), chelerythrine also suppressed zoosporogenesis which indicated the involvement of PKC in the process of zoospore release from sporangia (Islam et al., 2011).

The experimental results reported in the present study do not clarify the precise mechanism involved but they point out that induction of ATPase activity in mitochondria and/or translocation/imbalance of cations in the cells might suppress zoosporogenesis and impair motility of the zoospores. Therefore, elucidation of the role of ATPase in the swimming pattern and motility of zoospores will obviously help to advance our understanding of the biology and pathogenicity of the peronosporomycete phytopathogens. In this study, some linear tetrahydrofurans such as (+)-homonactic acid and bonactin also impaired motility of zoospores qualitatively and quantitatively similar to the macrotetrolide antibiotics. Therefore, naturally occurring low molecular weight inducers of ATPase might have high potential as lead compounds for designing novel effective agrochemicals against the peronosporomycete phytopathogens.

One of the important findings of our study is that the zoospores of a biotrophic phytopathogen, *P. viticola* halted by macrotetrolide antibiotics or gramicidin or nigericin were rapidly immobilized (Figures 2, 3B). A similar phenomenon was observed when zoospores of *P. capsici* were treated with higher concentration of dinactin and trinactin. In an earlier study, Graven et al. (1966) reported that nonactin, monactin, dinactin, trinactin and gramicidin induced swelling of mitochondria through induction of alterations in the ion translocation system. Nigericin has also been found to inhibit mitochondrial respiration by blocking the uptake of both K^+^ and inorganic phosphorus (Pi) ions (Graven et al., 1967). Therefore, rapid lysis of halted *P. viticola* zoospores by the macrotetrolides, gramicidin, or nigericin might be associated with alteration in the ion translocation system in the mitochondria of zoospores and/or imbalance of osmotic balance in the zoospores. It appears from this study that inhibition of mitochondrial respiration by any chemical inhibitor might impair motility of zoospores. Experiments using gramicidin and nigericin ionophores, Appiah et al. (2005) demonstrated that altering potassium homeostasis during zoospore swimming significantly influenced speed, swimming pattern, and encystment of zoospores of *Phytophthora* and *Pythium* species (Appiah et al., 2005). Although, almost all *P. viticola* zoospores stopped by nactic acids or macrotetrolides were lysed, the stopped *A. cochlioides* zoospores by the same compounds became, however, round cystospores instead of lysing. *A. cochlioides* is a soilborne phytopathogen and hence sensitivity of zoospores to surrounding complex heterogenous signals in soils might be different compared to a biotrophic leaf pathogen such as *P. viticola*. In soilborne pathogens, those zoospores failing to find their host during their motile stage or exposed to toxic substances, rapidly become cystospores by developing a cell wall (Islam, 2011). These cystospores can regenerate secondary zoospores under favorable conditions to search the host plant. This adaptive strategy may be absent or not essential in the leaf pathogen *P. viticola* and zoospores released on a leaf can easily find stomata for infection, and hence zoospores halted by nactic acids and macrotetrolides are immediately lysed. Motility inhibition of *A. cochlioides* zoospores without lysis by a nonhost metabolite, nicotinamide has been reported (Islam et al., 2004).

Nonactin, dinactin, trinactin and teranactin isolated from a variety of *Streptomyces* species are cyclotetralactones derived from nonactic acid and homononactic acid as building units of ionophoric character. Nonactin and its homologs such as monactin, dinactin, trinactin, and tetranactin were isolated as bioactive compounds from *Streptomyces* spp. by many researchers (Beck et al., 1962; Dominguez et al., 1962; Meyers et al., 1965; Haneda et al., 1974; Callewaert et al., 1988; Sobolevskaya et al., 2004; Hashida et al., 2012). The precursor of nonactin and other marcotetrolides, nonactic acid is biosynthesized in *Streptomyces* spp. by a type II polyketide synthase (PKS) system (Walczak et al., 2000). In our study, both cyclotetralactones and their precursor, nonactic acid and homononactic acids displayed inhibitory effects against zoosporogenesis and motility of zoospores but in varying concentrations. Among the nactic acids, the homonactic acid showed a higher activity than nonactin. A further study is needed to establish precise structure-activity relationships of these bioactive compounds which might lead to the development of an effective biopesticide against the peronosporomycete phytopathogens.

Zoosporogenesis and motility of zoospores are two life stages critically important for disease cycles and also virulence of the peronosporomycete phytopathogens (Latijnhouwers et al., 2004; Judelson and Blanco, 2005; Islam et al., 2011). A motility inhibitory compound, staurosporine has recently been found to successfully suppress development of downy mildew on treated grapevine leaves (Islam et al., 2011). Macrotetrolide antibiotics exhibit a very wide range of effects, ranging from antimicrobial to insecticidal, acaricidal, anticancer, antiprotozoan (coccidiostatic), immunosuppressive and antiparasitic (antihelminthic) (Meyers et al., 1965; Borrel et al., 1994; Zizka, 1998; Kusche et al., 2009). Plant growth promotion and exhibition of specific insecticidal effects of precursors of macrotetrolide antibiotics, nonactic and homononactic acids have been reported (Jizba et al., 1992). The zoospore motility inhibitory substances found in this study such as dinactin, and nactic acids produced by several *Streptomyces* spp. including a marine strain Act 8970 might help us to design strategies for biorational management of the notorious Peronosporomycete phytopathogens. This study reveals that macrotetrolide antibiotics are not only potential candidates for development of antiperonosporomycete agrochemicals but could also be used as tools for dissecting underlying molecular mechanisms governing zoosporogenesis and motility functions of zoospores of the fungus-like peronosporomycetes.

## Author contributions

MTI: Involved in conceived idea, designed and executed experiments, analyzed data and writing manuscript; AvT and HL: Involved in conceived idea, designed experiments, and critically edited manuscript.

### Conflict of interest statement

The authors declare that the research was conducted in the absence of any commercial or financial relationships that could be construed as a potential conflict of interest.
